# Involvement of *CCL2* and *CH25H* Genes and TNF signaling pathways in mast cell activation and pathogenesis of chronic spontaneous urticaria

**DOI:** 10.3389/fimmu.2023.1247432

**Published:** 2023-08-14

**Authors:** Xiaobin Fang, Yueyi Weng, Xiaochun Zheng

**Affiliations:** ^1^ Department of Anesthesiology/Critical Care Medicine, Shengli Clinical Medical College of Fujian Medical University, Fujian Provincial Key Laboratory of Critical Care Medicine, Fujian Provincial Hospital, Fuzhou, Fujian, China; ^2^ Department of Anesthesiology, Fujian Provincial Hospital, Shengli Clinical Medical College of Fujian Medical University & Fujian Emergency Medical Center, Fujian Provincial Key Laboratory of Emergency Medicine, Fuzhou, Fujian, China; ^3^ Fujian Provincial Key Laboratory of Critical Medicine, Fuzhou, Fujian, China; ^4^ Fujian Provincial Co-constructed Laboratory of “Belt and Road”, Fuzhou, Fujian, China

**Keywords:** chronic spontaneous urticaria, cholesterol 25-hydroxylase, chemokine ligand 2, tumor necrosis factor, mast cells

## Abstract

Chronic spontaneous urticaria (CSU), a mast cell-driven disease, substantially affects the quality of life. While genetics affect CSU susceptibility and severity, the specific genetic factors associated with mast cell activation in CSU remain elusive. We aimed to identify key genetic factors and investigate their roles in CSU pathogenesis. Two gene expression datasets from the Gene Expression Omnibus were merged and validated using principal component analysis and boxplots. The merged dataset was subjected to limma and weighted gene co-expression network analyses. Genes whose expression correlated highly with CSU were identified and analyzed using Gene Set Enrichment Analysis (GSEA), Gene Ontology (GO), and Kyoto Encyclopedia of Genes and Genomes (KEGG) enrichment analyses. As GSEA, GO, and KEGG analyses highlighted the importance of chemokine (C-C motif) ligand 2 (CCL2) and cholesterol 25-hydroxylase (CH25H) gene and tumor necrosis factor (TNF) signaling pathways in CSU; the three corresponding genes were knocked down in human mast cell line-1 (HMC-1), followed by incubation with thrombin to mimic CSU pathogenesis. CCL2, CH25H, and TNF knockdown reduced excitability and cytokine production in HMC-1. Our findings suggest that genes involved in the CCL2, CH25H, and TNF pathways play crucial roles in CSU pathogenesis, providing insights into potential therapeutic targets for CSU treatment.

## 1 Introduction

Chronic spontaneous urticaria (CSU), a skin disease driven by mast cells, is characterized by the recurrence of transient wheals, angioedema, or both for more than 6 weeks (1). This condition significantly impacts the quality of life of patients, causing physical discomfort, emotional distress, sleep disturbances, and social isolation due to its debilitating symptoms. Furthermore, CSU imposes a substantial burden on society, as it leads to increased healthcare costs, reduced work productivity, and absenteeism (2, 3). Although progress has been made in understanding the pathogenesis of CSU, the exact mechanisms underlying this disease, particularly those related to its recurrence and susceptibility, are not completely understood. Recent research suggests that genetic factors may play a crucial role in the development and recurrence of CSU (4). However, the specific genetic mechanisms underlying this process remain unclear.

Investigating the genetic factors underlying CSU requires a large sample size to ensure reliability; however, individual studies may struggle to obtain a substantial number of samples. The Gene Expression Omnibus (GEO, http://www.ncbi.nlm.nih.gov/geo/) addresses this issue by serving as a free public repository for high-throughput functional genomic data, allowing researchers to merge multiple studies, increase sample sizes, compare datasets, and gain insights into the gene expression patterns of a disease (5). In this context, bioinformatics tools such as ‘limma’ (6) and weighted gene co-expression network analyses (WGCNA) (7) have been widely used to identify relevant genes and molecular pathways.

Mast cells are immune cells that play a central role in the pathogenesis of CSU. Activation of mast cells leads to the production and release of multiple inflammatory mediators, such as histamine, cytokines, and chemokines. These mediators are responsible for the characteristic symptoms of CSU, including wheals, itching, and angioedema (8, 9). Moreover, one well-established mechanism in CSU pathogenesis involves thrombin, a serine protease involved in blood coagulation, which activates mast cells and promotes the release of inflammatory mediators (10, 11). Additionally, human mast cell line (HMC-1) is a well-established and widely used model that exhibits many characteristics of mast cells, making them a valid tool for studying mechanisms of mast cells in CSU (12). Furthermore, histamine (13), a key player in CSU, along with other inflammatory mediators including chemokine (C-C motif) ligand (CCL) 2, C-X-C motif chemokine ligand (CXCL)1, CXCL5, interleukin (IL) 6, tumor necrosis factor (TNF)-α, and vascular endothelial growth factor (VEGF) were found to be essential for CSU severity (14–21). Therefore, monitoring the production of these mediators in the thrombin-activated HMC-1 model may help us evaluate the severity of CSU.

In this study, we aimed to investigate gene expression patterns in CSU using the publicly available GEO database. Bioinformatics analyses were conducted on merged datasets using the limma and WGCNA packages to identify key genes and pathways associated with the disease. To validate these findings, experiments were performed using HMC-1 cell and the effects of key genes and pathways on mast cell-derived inflammatory cytokine production was investigated following the knockdown of these genes or channels and co-incubation with thrombin. This study will contribute to a better understanding of the genetic factors involved in CSU and may uncover potential therapeutic targets for this debilitating condition.

## 2 Materials and methods

**Figure 1:**
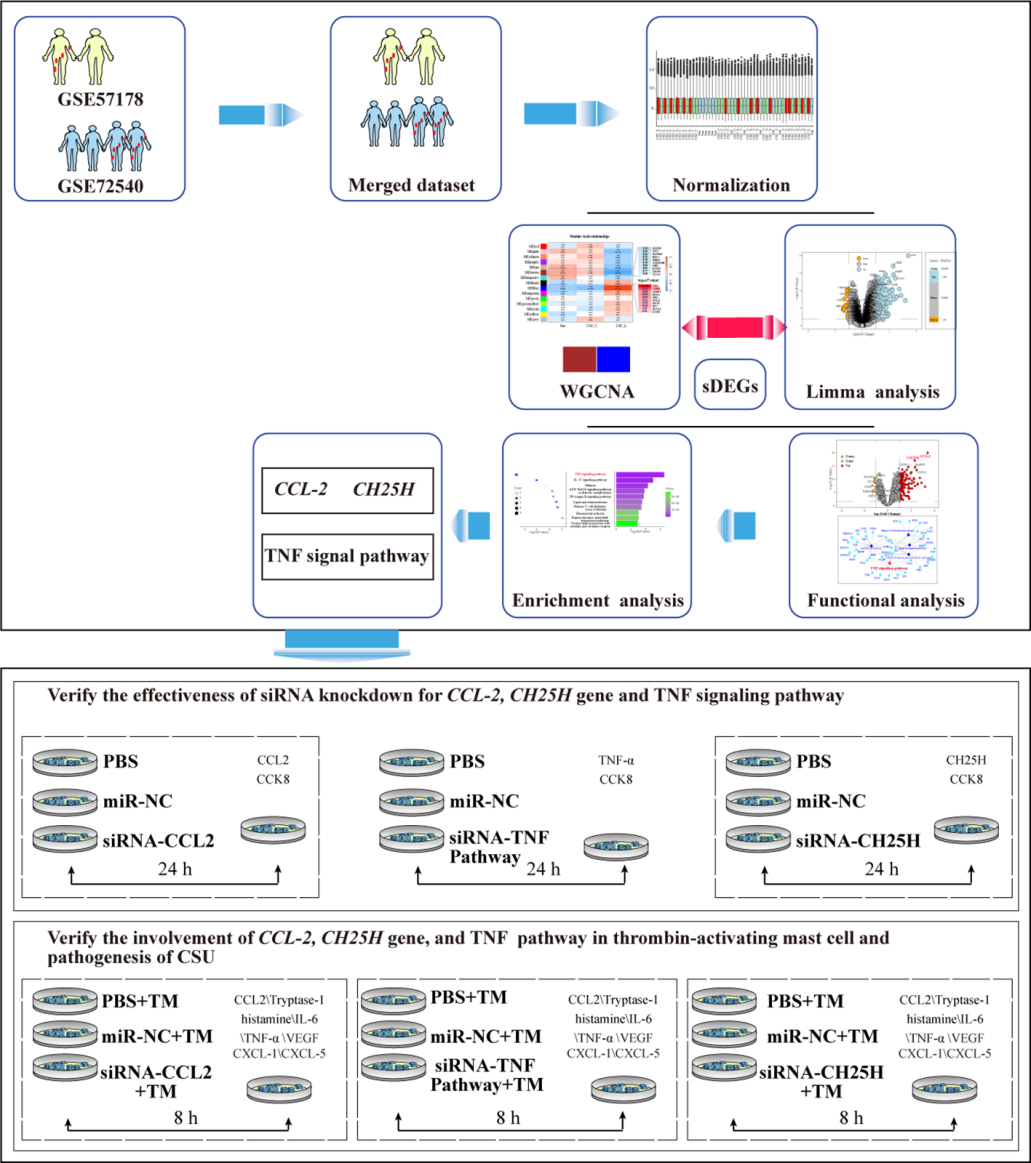
Flowchart of the study. PCA, principal component analysis; WGCNA, weighted gene co-expression network analysis; DEGs, differentially expressed genes; sDEGs, specific differentially expressed genes; TM, thrombin 0.2 U/mL.

A flowchart of the process is shown in **Figure 1**.

### 2.1 Integrating and validating CSU microarray data from GEO

Human CSU-related gene expression data was searched in the GEO database of NCBI (https://www.ncbi.nlm.nih.gov/geo/) with “urticaria” and “*Homo sapiens*” as keywords. Microarray data from the same platform were selected and screened. Eligible microarray data, specifically GSE57178 and GSE72540, were merged based on gene names, and the SVA R package was used for normalization and batch effect removal. GSE57178 and GSE72540 both included skin samples from three groups: lesional (CSU-L) and non-lesional skin (CSU-C) of patients with active CSU, as well as from healthy volunteers (Nor). The adjusted dataset was validated using boxplot and principal component analysis (PCA) to confirm the absence of confounding batch-variable effects. This step was crucial to ensure that the merged dataset was appropriate for subsequent analyses. The normalized dataset was subjected to limma analysis and WGCNA.

### 2.2 Limma analysis and WGCNA

Limma analysis was performed using the ‘limma’ package in R software. A design matrix that included the experimental conditions (Nor, CSU-C, and CSU-L) as covariates was created. The differentially expressed genes (DEGs) among the Nor, CSU-C, and CSU-L groups were estimated using the empirical Bayes method. The average expression, *P*-values, adj. *P*-val, and logFC were calculated for each gene. The purpose of the limma analysis was to identify and quantify the DEGs between the CSU-L and the other groups (Nor and CSU-C).

The top 5,000 genes with the highest median absolute deviation in the merged dataset were selected for WGCNA. The WGCNA package for R identified the co-expressed gene modules and explored the correlation between the gene network and CSU-L. The soft power threshold was selected using the pickSoftThreshold function. Co-expression modules were identified using the blockwiseModules function with modified parameters, including soft threshold (power), minModuleSize (22), and mergeCutHeight (0.25). A topological overlap matrix was used to evaluate network connectivity, and hierarchical clustering was used to construct topological overlap matrix dendrograms. Genes were clustered into different modules based on their expression patterns, and intermodule correlations were calculated and visualized using a heat map. Module-trait correlations were evaluated to determine the modules associated with CSU-L for analysis. The purpose of WGCNA was to identify gene modules co-expressed in CSU-L and explore their correlation with the disease state.

### 2.3 Identifying specific differentially expressed genes (sDEGs) in CSU-L using WGCNA and limma

The color modules that were significantly correlated with CSU-L were identified using WGCNA. Genes within these modules were selected for further analysis. The selected genes were paired with DEGs obtained from the limma analysis to generate an expression matrix for highly sDEGs. A volcano plot visually represented the filtering process, clearly displaying sDEGs associated with CSU-L meeting the criteria of *P* < 0.01 and the log FC cutoff (>1-fold upregulation or downregulation). The sDEGs were then subjected to GSEA and enrichment using GO and KEGG analyses to identify the specific signaling pathways associated with CSU. The aim of combining WGCNA and limma analysis was to pinpoint sDEGs and their associated pathways in CSU-L.

### 2.4 siRNA transfection and thrombin co-incubation

The reagents and cell information are provided in **Supplementary Material (Supplementary Data 1)**. Detailed methods for HMC-1 cell culture and the CCK-8 cell viability assay are provided in **Supplementary Material (Supplementary Data 2)**. Detailed protocols for quantitative polymerase chain reaction (qPCR), western blotting, and enzyme-linked immunosorbent assay (ELISA) are provided in **Supplementary Material (Supplementary Data 3)**. The primer sequences used are listed in **Supplementary Material (Supplementary Table 1)**.

siRNA Screening: HMC-1 cells were seeded and transfected with three distinct siRNAs against CCL2, cholesterol 25-hydroxylase (CH25H), and TNF using Lipofectamine 3000 transfection reagent (Invitrogen, Waltham, MA, USA) according to the manufacturer’s instructions. The NC group was transfected with non-sense siRNA, while the CON group consisted of HMC-1 cells cultured with an equal volume of PBS as a control. After 48 h, the transfection efficiency was assessed using qPCR to determine the knockdown of *CCL2*, *CH25H*, and *TNF* mRNA levels, and the most effective siRNA was selected for each target. The siRNA sequences are presented in **Supplementary Material (Supplementary Table 2)**.

Thrombin co-incubation: HMC-1 cells were transfected with CCL2, CH25H, and TNF siRNAs, NC siRNA, or CON. After 48 h, the transfected cells were co-incubated with thrombin for 8 or 16 h. Cytokine mRNA levels were assessed using qPCR and western blotting, and protein levels were measured using ELISA.

### 2.5 Statistical analysis

Data are presented as means ± standard deviations. Data analysis was performed using SPSS v20.0 (SPSS Inc., Chicago, IL, USA). CCK-8 data were analyzed using one-way analysis, whereas data regarding the expression of multiple inflammatory mediators in HMC-1 cells and supernatants were analyzed using a one-way analysis of variance with Dunnett’s multiple comparison tests. *P-*values less than 0.05 were considered significant.

## 3 Results

### 3.1 GSE57178 and GSE72540 were merged into a single dataset for further analysis

Microarray data from GSE57178 (23) and GSE72540 (24) were used for the analysis. These datasets were chosen based on their use of the same sequencing platform and consistent sample classification, ensuring compatibility and reliability for comparative analysis. GSE57178 included six skin samples from CSU-L, seven skin samples from the CSU-C of patients with active CSU, and five skin samples from Nor. GSE72540 included 10 skin samples from CSU-L, 14 skin samples from CSU-C of the same patients with severely active CSU, and 7 skin samples from Nor. Patients in both studies were not receiving any other treatments at the time of sample collection. Both datasets were based on the GPL16699 platform (Agilent-039494 SurePrint G3 Human GE v2 8×60K Microarray 039381; Agilent Technologies, Santa Clara, CA, USA). A single matrix was obtained by merging GSE57178 and GSE72540 for further analysis while preserving the matched gene symbols, combined gene expression matrix, and group information. A flowchart of the study is presented in **Figure 1**.

### 3.2 Post-correction boxplots and PCA plots showed consistent biology-driven clustering

**Figure 2:**
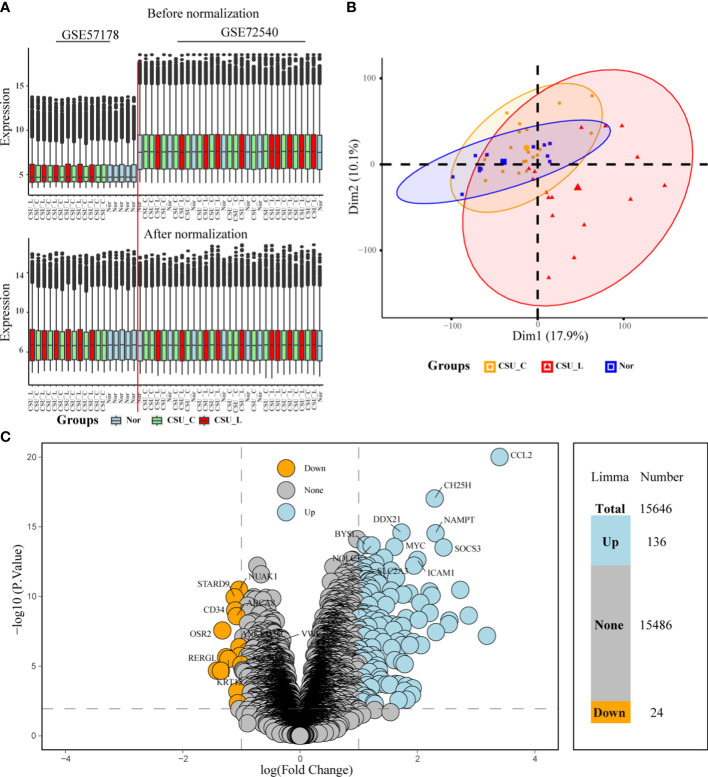
Post-correction boxplots and PCA plots showing consistent biology-driven clustering. **(A)** Post-correction boxplots of merged dataset consistency. **(B)** PCA results. **(C)** Volcano plot showing differentially expressed genes from limma analysis. Arabic numerals represent the number of up-, non-, and downregulated genes based on *P* < 0.01, logFC cutoff >1-fold upregulation or downregulation. PCA, principal coordinate analysis.

Upon batch-effect correction, the boxplots exhibited more uniform and comparable distributions of gene expression values across all sample groups (**Figure 2A**). Moreover, post-batch effect correction clustered the samples more closely based on their respective biological groups (CSU-L, CSU-C, and Nor) (**Figure 2B**). This suggests that the batch effect was successfully mitigated, rendering the merged dataset more appropriate for subsequent analyses.

### 3.3 Limma analysis and WGCNA: uncovering significant gene modules and sDEGs in CSU-L subtype

Limma analysis generated a table of DEGs with associated statistics focusing on their relationship with the CSU-L group. The table includes log fold change (logFC), average expression, t-statistic (t), *P*-value, adj. *P*-val, and gene names. Of the 15,646 genes, 136 were upregulated and 24 were downregulated (*P* < 0.01, logFC cutoff >1-fold upregulation or downregulation) (**Figure 2C**). The cutoff criteria (*P* < 0.01, logFC > 1-fold upregulation or downregulation) were selected based on their ability to effectively reduce false positives while maintaining a sufficient number of biologically relevant genes for subsequent enrichment analysis and experimental validation.

**Figure 3:**
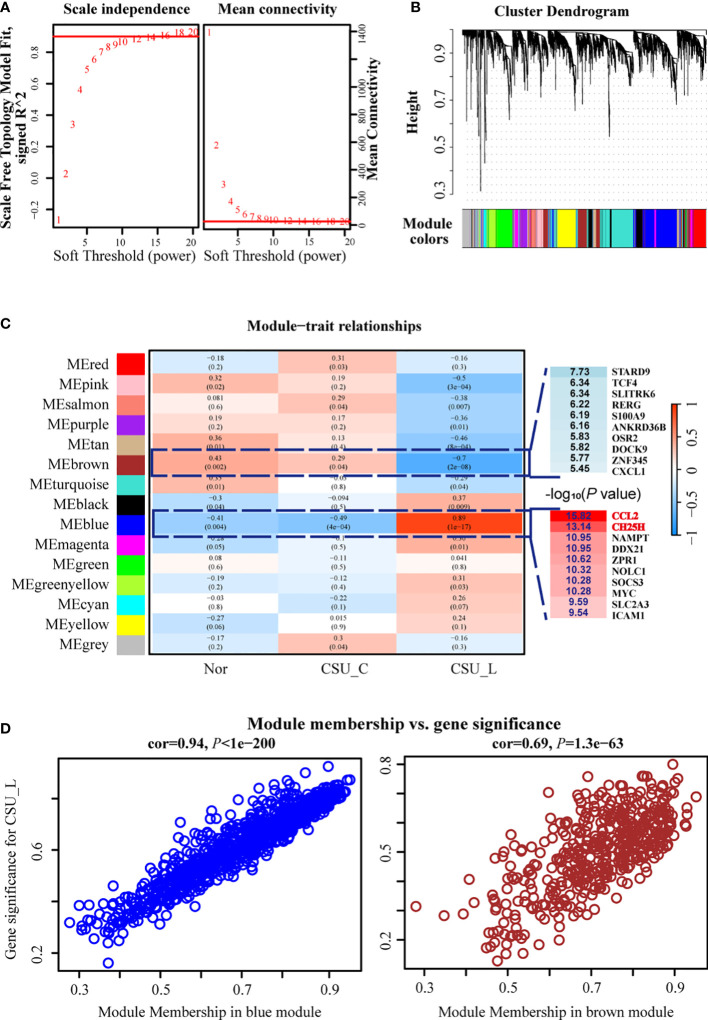
WGCNA revealed strong correlations between the blue and brown modules, particularly the blue module and the CSU-L subtype. **(A)** Optimal soft threshold. **(B)** Genes were grouped into 15 distinct modules. **(C)** Blue and brown modules were closely associated with the CSU-L subtype. **(D)** Correlation of the blue and brown modules with CSU. CSU, chronic spontaneous urticaria.

The optimal soft threshold for a scale-free network in WGCNA was determined to be 10. **Figure 3A** shows the scale-free topology model fit (signed R^2^) and mean connectivity as functions of the soft threshold values, confirming a chosen power of 10. Genes were grouped into 15 distinct modules *via* hierarchical clustering based on topological overlaps, representing gene clusters with similar expression patterns (**Figure 3B**).

The module-trait relationship heat map revealed strong correlations between the blue and brown modules, particularly the blue module and the CSU-L subtype, suggesting their potential significance in CSU-related biological processes (**Figure 3C**). Notably, the *CCL2* gene within the blue module emerged as the most significant DEG associated with CSU-L, while the CH25H gene was the second most significant DEG (**Figure 3C**). The blue module showed a strong correlation with CSU (COR = 0.94, *P* < 1e-200), whereas the brown module exhibited a significant but weaker association (COR = 0.69, *P* = 1.3e-63) (**Figure 3D**).

The blue and brown modules contain 846 and 441 genes, respectively. These genes were subjected to DEG analysis using limma, yielding a dataset of highly sDEGs.

### 3.4 Functional analysis of sDEGs: highlighting the potential importance of *CCL2*, *CH25H*, and TNF signaling pathways in CSU-L

**Figure 4:**
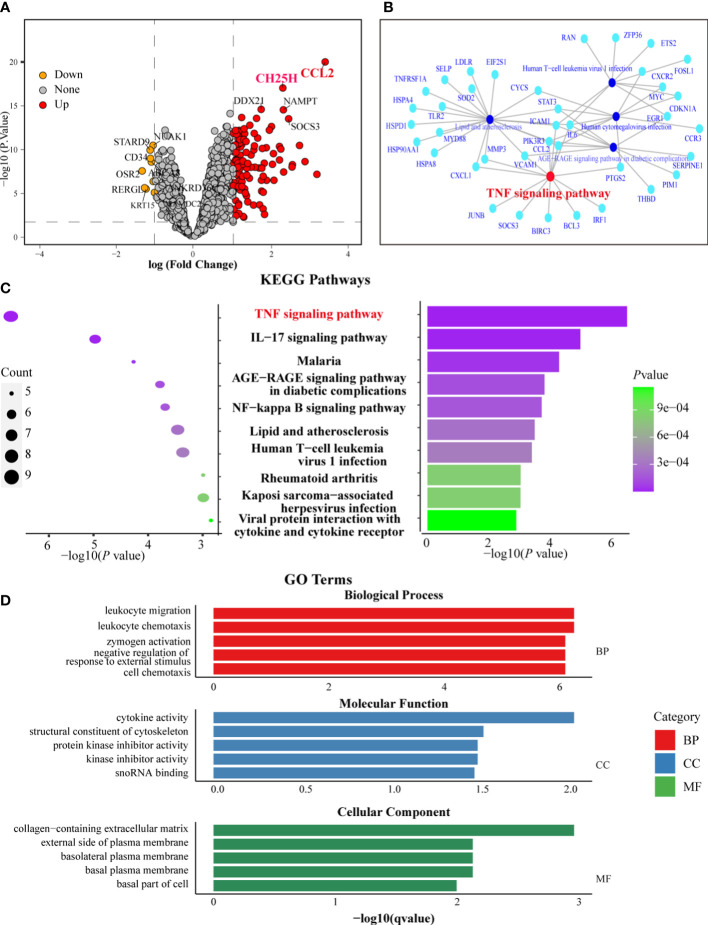
Functional analysis of sDEGs highlighted the CCL-2 and TNF signaling pathways in CSU-L. **(A)** Volcano plot showing sDEGs. **(B)** GSEA highlighting the TNF signaling pathway. **(C)** KEGG enrichment analysis highlighting the TNF signaling pathway. **(D)** Results of GO enrichment analysis.

Based on *P* < 0.01 and logFC cutoff (>1-fold up or downregulation), 110 genes were upregulated, and 9 genes were downregulated among sDEGs. Notably, the *CCL2* gene had the highest logFC and -log *P*-value, indicating its close association with CSU-L (**Figure 4A**). The *CH25H* gene, however, also exhibited a high logFC and -log *P*-value, making it the second most closely associated gene with CSU-L (**Figure 4A**).

GSEA of sDEGs identified 25 enriched pathways (adjusted *P*-value < 0.05), with the “TNF signaling pathway” among the top pathways, emphasizing its potential role in CSU-L. Other significant pathways included “lipid metabolism and atherosclerosis,” “AGE-RAGE signaling pathway in diabetic complications,” and “human cytomegalovirus infection” (**Figure 4B**). In the over-representation test of sDEGs using KEGG pathways, the “TNF signaling pathway” was again among the top enriched terms, further reinforcing its importance in the context of CSU. Additional pathways included “IL-17 signaling pathway,” “malaria,” and “AGE-RAGE signaling pathway in diabetic complications” (**Figure 4C**).

GO enrichment analysis of sDEGs revealed significant terms in three categories: biological process, molecular function, and cellular components. The key biological process terms included “leukocyte migration and chemotaxis,” “zymogen activation,” and “negative regulation of responses to external stimuli.” For molecular function, the top terms were “cytokine activity,” “cytoskeletal structural constituents,” and “protein kinase inhibitor activity.” In cellular components, the most enriched terms were “extracellular matrix” and “plasma membrane components” (**Figure 4D**). The *CCL2* and *CH25H* genes and TNF signaling pathways were also associated with biological process of “leukocyte migration and chemotaxis,” “negative regulation of response to external stimulus,” and “cell chemotaxis,” as well as the molecular function of “cytokine activity.”

Considering the significant roles of the *CCL2* and *CH25H* genes and TNF signaling pathways revealed through the functional analysis of sDEGs, we further investigated their involvement in CSU using cellular experiments.

### 3.5 siRNA-mediated *CCL2* and *TNF* knockdown mitigated mast cell activation and CSU pathogenesis

**Figure 5:**
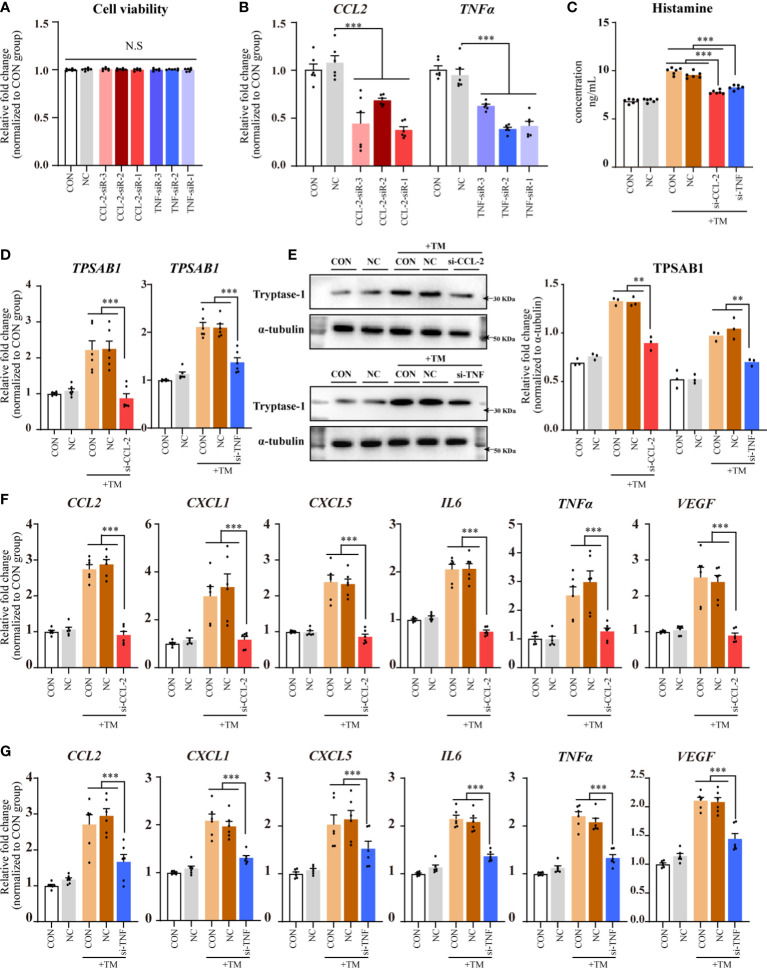
CCL-2 and TNF gene knockdown using siRNAs reduced mast cell activation and CSU pathogenesis. Data from 2–5 independent trials. One-way ANOVA, followed by Tukey’s multiple comparisons test, was used for group comparisons **P < 0.01; ***P < 0.001; N.S, no statistical difference. **(A)** CCK8 assay results. **(B)** qPCR for HMC-1 cells transfected with CCL-2 or TNF siRNA. **(C)** ELISA for HMC-1 supernatant transfected with CCL-2 or TNF siRNA following thrombin incubation. **(D)** qPCR of HMC-1 cells with CCL-2 or TNF siRNA transfection following thrombin incubation. **(E)** Western blot analysis. (Left) Representative images; (Right) Densitometric quantification using ImageJ software. **(F, G)** qPCR results for HMC-1 cells with CCL-2 or TNF siRNA transfection following thrombin incubation. TM, thrombin 0.2 U/mL.

The results of the cell counting kit (CCK)-8 assay did not show any significant differences in cell viability among the transfected groups (CCL2 and TNF siRNAs) compared with that of NC and CON groups, indicating that transfection did not negatively affect HMC-1 cell viability (**Figure 5A**).

qPCR analysis revealed a significant knockdown of *CCL2* and TNF in HMC-1 cells transfected with their respective siRNAs compared with those in the NC group. The most effective siRNAs against CCL2 and TNF were selected for further experiments (**Figure 5B**).

Compared with that in the NC and CON groups, the transfected groups (CCL2 and TNF siRNAs) exhibited reduced histamine levels in the supernatants (**Figure 5C**) and significantly decreased *TPSAB1* mRNA expression in HMC-1 cells (*P* < 0.01; **Figure 5D**). Western blot analysis revealed that tryptase-1 levels decreased in the transfected groups (CCL-2 and TNF siRNAs) (**Figure 5E**). Since tryptase-1 is a specific marker of mast cell activation, and histamine is a major pathogenic factor in CSU, these results suggest that *CCL2* and *TNF* knockdown with the corresponding siRNAs reduces mast cell activation and CSU pathogenesis.

Compared with that in the NC and CON groups, the transfected groups (CCL2 and TNF siRNAs) displayed decreased expression of *CCL2*, *CXCL1*, *CXCL5*, *IL6*, *TNFα*, and *VEGF* mRNAs in HMC-1 cells (**Figures 5F, G**).

### 3.6 siRNA-mediated downregulation of the *CH25H* gene attenuates mast cell activation and CSU pathogenesis

**Figure 6:**
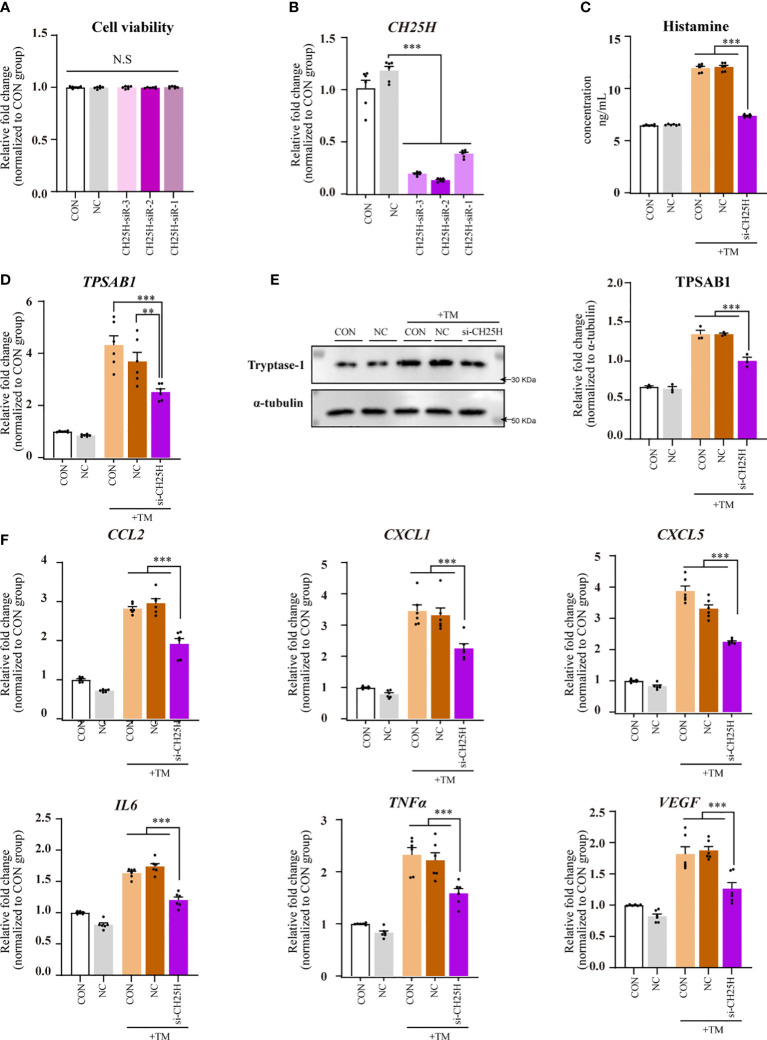
CH25H gene knockdown using siRNAs reduced mast cell activation and CSU pathogenesis. Data were from 2–5 independent trials. One-way ANOVA, followed by Tukey’s multiple comparisons test, was used for group comparisons. * indicates a statistical difference between groups. **P < 0.01; ***P < 0.001; N.S, no statistical difference. **(A)** CCK8 assay results. **(B)** qPCR for HMC-1 cells transfected with CH25H siRNA. **(C)** ELISA for HMC-1 supernatant transfected with CH25H following thrombin incubation. **(D)** qPCR results for HMC-1 cells with CH25H siRNA transfection following thrombin incubation. **(E)** Western blot analysis. (Left) Representative western blot images; (Right) Densitometric quantification using ImageJ software. **(F)** qPCR results for HMC-1 cells with CH25H siRNA transfection following thrombin incubation. TM, thrombin 0.2 U/mL.

CCK-8 assay results showed no significant differences in cell viability among the transfected CH25H siRNAs groups compared with that of the NC and CON groups, indicating that transfection did not negatively affect HMC-1 cell viability (**Figure 6A**).

Quantitative PCR analysis revealed a significant knockdown of *CH25H* in HMC-1 cells transfected with CH25H siRNAs compared with those in the NC group. The most potent CH25H-targeting siRNAs were chosen for subsequent experiments (**Figure 6B**).

A reduction in histamine levels in the supernatants was observed in the CH25H siRNA-transfected group compared with the NC and CON groups (**Figure 6C**). This group also exhibited significantly diminished *TPSAB1* mRNA expression in HMC-1 cells (*P* < 0.01; **Figure 6D**). Western blot analysis revealed that tryptase-1 levels decreased in the CH25H siRNAs transfected groups (**Figure 6E**). These results imply that *CH25H* knockdown with the corresponding siRNAs mitigates mast cell activation and CSU pathogenesis.

Compared with the NC and CON groups, the group transfected with CH25H siRNAs showed decreased expression of *CCL2*, *CXCL1*, *CXCL5*, *IL6*, *TNFα*, and *VEGF* mRNAs in HMC-1 cells (**Figure 6F**).

## 4 Discussion

In this study, we showed that the *CCL2* and *CH25H* genes and TNF signaling pathways play crucial roles in CSU development. Using comprehensive bioinformatics analyses, we initially identified key genetic factors and signaling pathways implicated in CSU pathogenesis. The *CCL2* and *CH25H* genes and TNF signaling pathways were screened using limma, WGCNA, GSEA, GO, and KEGG enrichment analyses. Furthermore, we confirmed their role in CSU by showing the reduced expression of mast cell activation marker tryptase-1, a crucial CSU mediator histamine, and several inflammatory mediators in HMC-1 cells transfected with siRNAs targeting the *CCL2* and *CH25H* genes and TNF pathways after thrombin incubation.

Merging of the GSE57178 and GSE72540 datasets was justified as their platforms and sample groups were shared. PCA and boxplot analyses demonstrated the effectiveness of the merged dataset in subsequent analyses. GSE57178 and GSE72540 were individually utilized in previous studies (25, 26). However, our dataset merging approach increased the sample size and enhanced reproducibility and data integration. Importantly, HMC-1 cell-based gene and signaling pathway knockdown experiments improved the reliability of the results of bioinformatics analysis (27), providing stronger evidence regarding the involvement of the *CCL2* and *CH25H* genes and TNF pathways in CSU in our study.

Combined analysis of limma and WGCNA showed that the *CCL2* gene, which had the greatest fold change and lowest *P*-value, and the *CH25H* gene, which was the second most significant, were both strongly associated with CSU, highlighting their potential relevance to the disease. ‘Limma’ is a R/Bioconductor package that uses linear models and empirical Bayesian methods to identify DEGs, estimate fold changes and *P*-values, and control false discovery rates (6), whereas WGCNA is a correlation-based network construction method that identifies co-expressed genes and modules correlated with a specific phenotype and can identify hub genes that play a central role in the network (7). Key genes identified by combining these methods increase the reliability and likelihood of their biological relevance to a disease (28). Both GSEA and KEGG enrichment analyses showed that the TNF signaling pathway was among the top enriched terms, indicating its critical role in CSU pathogenesis. GSEA and KEGG pathway analyses were used to identify key signaling pathways associated with certain diseases, providing valuable insights regarding the underlying molecular mechanisms and identifying potential therapeutic targets (29). The *CCL2* and *CH25H* genes and TNF signaling pathways were also highlighted in the results of GO enrichment analyses. Based on our findings, we suggest that the *CCL2* and *CH25H* genes and TNF signaling pathways are closely implicated in CSU pathogenesis.

The diminished production of tryptase-1, a unique marker of activated mast cells (30), in HMC-1 cells transfected with siRNAs targeting the *CCL2* and *CH25H* genes and TNF signaling pathways following thrombin incubation emphasizes their vital role in thrombin-mediated mast cell activation. Decreased secretion of histamine, a critical mediator of CSU (13), further indicates the involvement of the *CCL2* and *CH25H* genes and TNF pathways in CSU pathogenesis. Mast cells are the central effector cells in CSU, and thrombin-activated mast cells represent one of the confirmed contributors to CSU pathogenesis (10, 11). Stimulation conditions and designated time points for sample collection were based on previous studies (22, 31). The downregulation of various inflammatory mediators after mast cell knockdown highlights the intricate and potent inflammatory properties of the *CCL-2* and *CH25H* genes and TNF pathways in CSU. Multiple mediators are produced in CSU. In the present study, we focused on the mediators essential for CSU chronicity and severity. For example, IL6 and TNFα play pivotal roles in inflammatory responses and correlate with disease activity in CSU (14, 15). Additionally, the level of VEGF, which induces vasopermeability, is elevated in CSU patients (16). Perivascular non-necrotizing cellular infiltration is a hallmark of CSU (17, 18), and CXCL-1, CXCL5, and CCL2 promote the recruitment of eosinophils, neutrophils, and lymphocytes (19–21). These results suggest that mast cell activation associated with the *CCL2* and *CH25H* genes and TNF pathways may exert multifaceted effects on CSU. Given that the symptoms of CSU, such as hives and angioedema, are largely driven by the release of histamine and other inflammatory mediators from activated mast cells, the observed decrease in mast cell activation and mediator production following siRNA-mediated knockdown of *CCL2, CH25H*, and *TNF* genes in our HMC-1 model suggests a potential mechanism by which these genes may contribute to CSU pathogenesis. Furthermore, the elevated expression of these genes in CSU-L skin samples from patients, as well as their association with key inflammatory pathways, underscores their potential relevance to the disease process in CSU. These findings indicate that targeting the *CCL2* and *CH25H* genes and TNF pathways may offer therapeutic potential for managing CSU.

*CCL2*, which was identified as a gene significantly associated with CSU in our study, recruits and activates leukocytes, including mast cells, at inflammation sites (32). The TNF signaling pathway is involved in mast cell activation (33). The prominence of the TNF signaling pathway in GSEA and KEGG analysis in our study further highlights its significance. Elevated CCL2 (34) and TNFα levels (35) in CSU patients support their potential roles in CSU pathogenesis. Our findings emphasize that CCL2 and TNF pathways are potential therapeutic targets for CSU. The *CH25H* gene has been characterized as exerting complex yet critical effects on immune cells (36); its significance has been demonstrated in skin inflammation (37) and irritant contact dermatitis (38). Based on our findings, the *CH25H* gene may play a role in mast cell activation and the pathogenesis of CSU, warranting further research in the future.

The limitations of this study include the need for animal model studies to confirm our *in vitro* findings. In addition, we only investigated the mechanism involving thrombin-activated mast cells in CSU because of its complex pathogenesis. Further studies should investigate other mechanisms, such as those involving exosomes (39).

In conclusion, our study identified *CCL2* and *CH25H* genes and TNF signaling pathways as key factors in CSU pathogenesis, with HMC-1 cell experiments confirming their involvement in mast cell activation and inflammatory mediator production. These findings provide valuable insights regarding the molecular mechanism of CSU and identify potential therapeutic targets. Further studies are required to confirm these results and investigate the roles of other genes and pathways in CSU pathogenesis.

## Data availability statement

The original contributions presented in the study are publicly available. This data can be found here: 10.6084/m9.figshare.23895807.

## Author contributions

Conceptualization: XF. Data curation: YW. Formal Analysis: XF, XZ. Funding acquisition: XZ. Investigation XF. Methodology: XF, XZ. Project administration: XF and YW. Resources: XF and YW. Supervision: XF, XZ, and YW. Validation: XF, XZ, and YW. Visualization: XF, XZ, and YW. Writing – original draft: XF. Writing – review and editing: XF, XZ, and YW. All authors contributed to the article and approved the submitted version

## References

[1] Zuberbier T Aberer W Asero R Abdul Latiff AH Baker D Ballmer-Weber B. The EAACI/GA^2^LEN/EDF/WAO guideline for the definition, classification, diagnosis and management of urticaria. Allergy (2018) 73(7):1393–414. doi: 10.1111/all.13397 29336054

[2] Staubach P Eckhardt-Henn A Dechene M Vonend A Metz M Magerl M. Quality of life in patients with chronic urticaria is differentially impaired and determined by psychiatric comorbidity. Br J Dermatol (2006) 154(2):294–8. doi: 10.1111/j.1365-2133.2005.06976.x 16433799

[3] Ertaş R Erol K Hawro T Yılmaz H Maurer M. Sexual functioning is frequently and markedly impaired in female patients with chronic spontaneous urticaria. J Allergy Clin Immunol Pract (2020) 8(3):1074–82. doi: 10.1016/j.jaip.2019.10.046 31751760

[4] Cildir G Toubia J Yip KH Zhou M Pant H Hissaria P. Genome-wide analyses of chromatin state in human mast cells reveal molecular drivers and mediators of allergic and inflammatory diseases. Immunity (2019) 51(5):949–65. doi: 10.1016/j.immuni.2019.09.021 31653482

[5] Barrett T Wilhite SE Ledoux P Evangelista C Kim IF Tomashevsky M. NCBI GEO: archive for functional genomics data sets–update. Nucleic Acids Res (2013) 41(D1):D991–5. doi: 10.1093/nar/gks1193 PMC3531084 23193258

[6] Smyth GK. Linear models and empirical bayes methods for assessing differential expression in microarray experiments. Stat Appl Genet Mol Biol (2004) 3(1):1–25. doi: 10.2202/1544-6115.1027 16646809

[7] Langfelder P Horvath S. WGCNA: an R package for weighted correlation network analysis. BMC Bioinf (2008) 9:559. doi: 10.1186/1471-2105-9-559 PMC2631488 19114008

[8] Greaves MW Wall PD. Pathophysiology of itching. Lancet (1996) 348(9032):938–40. doi: 10.1016/S0140-6736(96)04328-0 8843816

[9] Church MK Kolkhir P Metz M Maurer M. The role and relevance of mast cells in urticaria. Immunol Rev (2018) 282(1):232–47. doi: 10.1111/imr.12632 29431202

[10] Kolkhir P Metz M Altrichter S Maurer M. Comorbidity of chronic spontaneous urticaria and autoimmune thyroid diseases: A systematic review. Allergy (2017) 72(10):1440–60. doi: 10.1111/all.13182 28407273

[11] Sakurai Y Morioke S Takeda T Takahagi S Hide M Shima M. Increased thrombin generation potential in patients with chronic spontaneous urticaria. Allergol Int (2015) 64(1):96–8. doi: 10.1016/j.alit.2014.07.006 25572563

[12] Butterfield JH Weiler D Dewald G Gleich GJ. Establishment of an immature mast cell line from a patient with mast cell leukemia. Leuk Res (1988) 12(4):345–55. doi: 10.1016/0145-2126(88)90050-1 3131594

[13] Guin JD. Treatment of urticaria. Med Clin North Am (1982) 66(4):831–49. doi: 10.1016/S0025-7125(16)31397-9 6178911

[14] Kasperska-Zajac A Sztylc J Machura E Jop G. Plasma IL-6 concentration correlates with clinical disease activity and serum C-reactive protein concentration in chronic urticaria patients. Clin Exp Allergy (2011) 41(10):1386–91. doi: 10.1111/j.1365-2222.2011.03789.x 21645137

[15] Hermes B Prochazka AK Haas N Jurgovsky K Sticherling M Henz BM. Upregulation of TNF-alpha and IL-3 expression in lesional and uninvolved skin in different types of urticaria. J Allergy Clin Immunol (1999) 103(2 Pt 1):307–14. doi: 10.1016/S0091-6749(99)70506-3 9949323

[16] Tedeschi A Asero R Marzano AV Lorini M Fanoni D Berti E. Plasma levels and skin-eosinophil-expression of vascular endothelial growth factor in patients with chronic urticaria. Allergy (2009) 64(11):1616–22. doi: 10.1111/j.1398-9995.2009.02069.x 19485983

[17] Batista M Calado R Gil F Cardoso JC Tellechea O Gonçalo M. Histopathology of chronic spontaneous urticaria with occasional bruising lesions is not significantly different from urticaria with typical wheals. J Cutan Pathol (2021) 48(8):1020–6. doi: 10.1111/cup.13985 33595130

[18] Giménez-Arnau AM DeMontojoye L Asero R Cugno M Kulthanan K Yanase Y. The pathogenesis of chronic spontaneous urticaria: the role of infiltrating cells. J Allergy Clin Immunol Pract (2021) 9(6):2195–208. doi: 10.1016/j.jaip.2021.03.033 33823316

[19] Shang Y Coppo M He T Ning F Yu L Kang L. The transcriptional repressor Hes1 attenuates inflammation by regulating transcription elongation. Nat Immunol (2016) 17(8):930–7. doi: 10.1038/ni.3486 PMC4955730 27322654

[20] Xu LL Warren MK Rose WL Gong W Wang JM. Human recombinant monocyte chemotactic protein and other C-C chemokines bind and induce directional migration of dendritic cells in vitro. J Leukoc Biol (1996) 60(3):365–71. doi: 10.1002/jlb.60.3.365 8830793

[21] Chang MS McNinch J Basu R Simonet S. Cloning and characterization of the human neutrophil-activating peptide (ENA-78) gene. J Biol Chem (1994) 269(41):25277–82. doi: 10.1016/S0021-9258(18)47243-2 7929219

[22] Fang X Liao R Yu Y Li J Guo Z Zhu T. Thrombin induces secretion of multiple cytokines and expression of protease-activated receptors in mouse mast cell line. Mediators Inflammm (2019) 2019:4952131. doi: 10.1155/2019/4952131 PMC6878808 31814803

[23] Patel OP Giorno RC Dibbern DA Andrews KY Durairaj S Dreskin SC. Gene expression profiles in chronic idiopathic (spontaneous) urticaria. Allergy Rhinol (Providence) (2015) 6(2):101–10. doi: 10.2500/ar.2015.6.0124 PMC4541630 26302730

[24] Giménez-Arnau A Curto-Barredo L Nonell L Puigdecanet E Yelamos J Gimeno R. Transcriptome analysis of severely active chronic spontaneous urticaria shows an overall immunological skin involvement. Allergy (2017) 72(11):1778–90. doi: 10.1111/all.13183 28407332

[25] Zhang T Feng H Zou X Peng S. Integrated bioinformatics to identify potential key biomarkers for COVID-19-related chronic urticaria. Front Immunol (2022) 13:1054445. doi: 10.3389/fimmu.2022.1054445 36531995 PMC9751185

[26] Peng S Zhang T Zhang S Tang Q Yan Y Feng H. Integrated bioinformatics and validation reveal IL1B and its related molecules as potential biomarkers in chronic spontaneous urticaria. Front Immunol (2022) 13:850993. doi: 10.3389/fimmu.2022.850993 35371000 PMC8975268

[27] Mohr S Bakal C Perrimon N. Genomic screening with RNAi: results and challenges. Annu Rev Biochem (2010) 79:37–64. doi: 10.1146/annurev-biochem-060408-092949 20367032 PMC3564595

[28] Yang Y Han L Yuan Y Li J Hei N Liang H. Gene co-expression network analysis reveals common system-level properties of prognostic genes across cancer types. Nat Commun (2014) 5:3231. doi: 10.1038/ncomms4231 24488081 PMC3951205

[29] Khatri P Sirota M Butte AJ. Ten years of pathway analysis: current approaches and outstanding challenges. PloS Comput Biol (2012) 8(2):e1002375. doi: 10.1371/journal.pcbi.1002375 22383865 PMC3285573

[30] Schwartz LB Min HK Ren S Xia HZ Hu J Zhao W. Tryptase precursors are preferentially and spontaneously released, whereas mature tryptase is retained by HMC-1 cells, Mono-Mac-6 cells, and human skin-derived mast cells. J Immunol (2003) 170(11):5667–73. doi: 10.4049/jimmunol.170.11.5667 12759448

[31] Fang X Li M Zhang W Li J Zhu T. Thrombin induces pro-inflammatory and anti-inflammatory cytokines secretion from human mast cell line (HMC-1) *via* protease-activated receptors. Mol Immunol (2022) 141:60–9. doi: 10.1016/j.molimm.2021.11.012 34808483

[32] Conti P Caraffa A Ronconi G Kritas SK Mastrangelo F Tettamanti L. Impact of mast cells in mucosal immunity of intestinal inflammation: inhibitory effect of IL-37. Eur J Pharmacol (2018) 818:294–9. doi: 10.1016/j.ejphar.2017.09.044 28970014

[33] Beaven MA. Our perception of the mast cell from Paul Ehrlich to now. Eur J Immunol (2009) 39(1):11–25. doi: 10.1002/eji.200838899 19130582 PMC2950100

[34] Santos JC de Brito CA Futata EA Azor MH Orii NM Maruta CW. Up-regulation of chemokine C-C ligand 2 (CCL2) and C-X-C chemokine 8 (CXCL8) expression by monocytes in chronic idiopathic urticaria. Clin Exp Immunol (2012) 167(1):129–36. doi: 10.1111/j.1365-2249.2011.04485.x PMC3248094 22132892

[35] Grieco T Porzia A Paolino G Chello C Sernicola A Faina V. IFN-γ/IL-6 and related cytokines in chronic spontaneous urticaria: evaluation of their pathogenetic role and changes during oMalizumab therapy. Int J Dermatol (2020) 59(5):590–4. doi: 10.1111/ijd.14812 32048727

[36] Blanc M Hsieh WY Robertson KA Kropp KA Forster T Shui G. The transcription factor STAT-1 couples macrophage synthesis of 25-hydroxycholesterol to the interferon antiviral response. Immunity (2013) 38(1):106–18. doi: 10.1016/j.immuni.2012.11.004 PMC3556782 23273843

[37] Takahashi H Nomura H Iriki H Kubo A Isami K Mikami Y. Cholesterol 25-hydroxylase is a metabolic switch to constrain T cell-mediated inflammation in the skin. Sci Immunol (2021) 6(64):eabb6444. doi: 10.1126/sciimmunol.abb6444 34623903 PMC9780739

[38] Fortino V Wisgrill L Werner P Suomela S Linder N Jalonen E. Machine-learning-driven biomarker discovery for the discrimination between allergic and irritant contact dermatitis. Proc Natl Acad Sci U.S.A. (2020) 117(52):33474–85. doi: 10.1073/pnas.2009192117 PMC7776829 33318199

[39] Fang X Li M He C Liu Q Li J. Plasma-derived exosomes in chronic spontaneous urticaria induce the production of mediators by human mast cells. J Invest Dermatol (2022) 142(11):2998–3008. doi: 10.1016/j.jid.2022.03.037 35659940

