# Supplementary material for: Involvement of CCL2 and CH25H Genes and TNF signaling pathways in mast cell activation and pathogenesis of chronic spontaneous urticaria
Source: Front Immunol. 2023 Aug 14;14:1247432. doi: 10.3389/fimmu.2023.1247432 (PMC10461452; doi:10.3389/fimmu.2023.1247432)
Supplement: Supplementary file 1 [file DataSheet_1.pdf]

SUPPLEMENTARY INFORMATION

Title: Involvement of CCL-2, CH25H Gene, and TNF Signaling Pathway in Mast Cell Activation and Pathogenesis of Chronic Spontaneous Urticaria

Authors: Xiaobin Fang, Yueyi Weng, Xiaochun Zheng*

Supplemental Data 1. Reagents and cells

HMC-1 cells were purchased from the Shanghai Cell Bank of the Chinese Academy of Sciences (Shanghai, China). The CCK-8 was obtained from Boster Biological Technology (Wuhan, China). ELISA kits for histamine, antibody against tryptase-1 and β-tubulin were purchased from Neobioscience (Shenzhen, China). CCL-2, CH25H, and TNF siRNAs were purchased from Tsingke Biotechnology Co., Ltd (Beijing, China). Other reagents were of analytical grade and were purchased from Sigma-Aldrich (St. Louis, MO, USA).

Supplemental Data 2. Cell culture and CCK-8 Cell Viability Assay

HMC-1 cells were cultured at 37°C in a 95% O2/5% CO2 atmosphere using IMDM (SH30228.01; Gibco) supplemented with 100 μg/mL streptomycin, 100 U/mL penicillin, and 10% heat-inactivated fetal bovine serum. Prior to experimentation, HMC-1 cells were grown in serum-free basal medium, washed twice with PBS, and provided with fresh IMDM (1 mL) in each culture dish.

CCK-8 Cell Viability Assay: To assess the effect of transfection on cell viability, a Cell Counting Kit-8 (CCK-8) assay was performed. HMC-1 cells were seeded in 96-well plates and transfected with the CCL-2, CH25H, and TNF siRNAs, as well as with the negative control (NC) and untreated control (CON) groups. After a 48-hour incubation period, 10 μl of CCK-8 solution was added to each well, and the plates were further incubated for 2 hours at 37°C. The absorbance at 450 nm was measured using a microplate reader to determine cell viability.

Supplemental Data 3.

qPCR: Total RNA was extracted using TRIzol reagent and reverse transcribed into cDNA using a reverse transcription kit (Bio-Rad Laboratories). cDNA expression was analyzed with Maxima SYBR Green qPCR Master Mix (Fermentas) following the manufacturer's instructions. Relative mRNA expression was normalized to 18S RNA. qPCR was employed to identify the most effective siRNA for each target gene and assess the expression of inflammatory mediators in thrombin-incubated HMC-1 cells.

Western blotting: Cells were collected, washed, and lysed in RIPA buffer. Protein concentrations were normalized using BCA assays. Samples were separated by electrophoresis and transferred to PVDF membranes. Membranes were blocked with 5% milk and incubated overnight at 4°C with human antibodies against lamin B1 and tryptase-1. Blots were developed with HRP-conjugated secondary antibodies and visualized. Protein expression was quantified relative to lamin B1 levels.

ELISA: Histamine concentrations in HMC-1 cell supernatants incubated with thrombin were measured using ELISA kits following the manufacturer's instructions. Mediator concentrations were expressed as ng/mL of protein.

Supplementary Table 1

The sequence of siRNA in our experiments.

Name	Sence(5'-3')	Anti-sence(3'-5')
Human-siCCL2-1	CAGCAGCAAGUGUCCCAAA	UUUGGGACACUUGCUGCUG
Human-siCCL2-2	UGUUAUAACUUCACCAAUA	UAUUGGUGAAGUUAUAACA
Human-siCCL2-3	CAAGAAUCAUUAAUACAAA	UUUGUAUUAAUGAUUCUUG
Human-siTNF-1	GUCAGAUCAUCUUCUCGAA	UUCGAGAAGAUGAUCUGAC
Human-siTNF-2	GACUCAGCGCUGAGAUCAA	UUGAUCUCAGCGCUGAGUC
Human-siTNF-3	CCUACCAGACCAAGGUCAA	UUGACCUUGGUCUGGUAGG
Human-siCH25H-1	GCUCCGUACUUUACACACU	AGUGUGUAAAGUACGGAGC
Human-siCH25H-2	CAGCAUGUGAUGUUUGUGU	ACACAAACAUCACAUGCUG
Human-siCH25H-3	GCUUCUUCGACAUGAUGAA	UUCAUCAUGUCGAAGAAGC

Supplementary Table 2

The primers used in our study.

primers	Forward	Reverse
Tryptase-1	GACAGGCTGGGGTAACATCG	GAAGGCAGAAGAATTGGGACTC
IL-6	CAATATTAGAGTCTCAACCCCCAA	TCACCAGGCAAGTCTCCTCA
TNF-α	CTCGAACCCCGAGTGACAAG	TGAGGTACAGGCCCTCTGAT
CCL-2	GATCTCAGTGCAGAGGCTCG	TTTGCTTGTCCAGGTGGTCC
CXCL-1	CGCCTCTGATCCAAGCCAC	CTGCACATAGTCCTGCACCA
CXCL-5	GCCTGTTCTAGTCCTGGTGG	GGCATCTAAAAAGCTCAGCAATG
VEGF	CTCACCAAGGCCAGCACATA	CCGGGATTTCTTGCGCTTTC
CH25H	TGCAACTTCGCTCCGTACTT	AAGGACACTTGGTATGCCC

The primers used for qPCR in our study.
